# Generalizing hydrogel microparticles into a new class of bioinks for extrusion bioprinting

**DOI:** 10.1126/sciadv.abk3087

**Published:** 2021-10-15

**Authors:** Shangjing Xin, Kaivalya A. Deo, Jing Dai, Navaneeth Krishna Rajeeva Pandian, David Chimene, Robert M. Moebius, Abhishek Jain, Arum Han, Akhilesh K. Gaharwar, Daniel L. Alge

**Affiliations:** 1 Department of Biomedical Engineering, Texas A&M University, College Station, TX 77843, USA.; 2 Department of Electrical and Computer Engineering, Texas A&M University, College Station, TX 77843, USA.; 3 Department of Medical Physiology, College of Medicine, Texas A&M Health Science Center, College Station, TX 77843, USA.; 4 Department of Cardiovascular Sciences, Houston Methodist Academic Institute, Houston, TX 77030, USA.; 5 Department of Materials Science and Engineering, Texas A&M University, College Station, TX 77843, USA.; 6 Center for Remote Health Technologies and Systems, Texas A&M University, College Station, TX 77843, USA.; 7 Interdisciplinary Graduate Program in Genetics, Texas A&M University, College Station, TX 77843, USA.

## Abstract

Hydrogel microparticles (HMPs) are an emerging bioink that can allow three-dimensional (3D) printing of most soft biomaterials by improving physical support and maintaining biological functions. However, the mechanisms of HMP jamming within printing nozzles and yielding to flow remain underexplored. Here, we present an in-depth investigation via both experimental and computational methods on the HMP dissipation process during printing as a result of (i) external resistance from the printing apparatus and (ii) internal physicochemical properties of HMPs. In general, a small syringe opening, large or polydisperse size of HMPs, and less deformable HMPs induce high resistance and closer HMP packing, which improves printing fidelity and stability due to increased interparticle adhesion. However, smooth extrusion and preserving viability of encapsulated cells require low resistance during printing, which is associated with less shear stress. These findings can be used to improve printability of HMPs and facilitate their broader use in 3D bioprinting.

## Teaser

Bioprinting hydrogel microparticles requires consideration of size, stiffness, and nozzle geometry due to jamming effects.

## INTRODUCTION

Three-dimensional (3D) bioprinting fabricates scaffolds and extracellular matrices with living cells and has potentials to meet the needs for tissue engineering (*1*–*4*). However, in extrusion bioprinting, a major category of 3D bioprinting, the number of bioink choices is limited because most soft biomaterials are unable to support subsequent printing layers without cross-linking to construct complex 3D structures. Meanwhile, with curing, they lose the extrudability and also result in high shear stress during printing, damaging the encapsulated cells. Various strategies have been developed to modify existing 3D printing techniques and expand the bioink toolkit, including locking the printed shape in an uncross-linked state using suspension baths (*5*, *6*), rapid improvement of the mechanical strength of bioinks after extrusion via double network formation (*7*), photopolymerization immediately before extrusion via transparent nozzles (*8*), and reinforcing the bioinks using rheological additives such as nanoparticles (*9*, *10*). These advanced printing methods have enabled fabrication of intricate 3D structures from many soft biopolymers. However, dedicated designs of cross-linking chemistries and suspension baths are required for these strategies, which limits their versatility to broad ranges of soft biomaterials of interest.

Densely packed or jammed hydrogel microparticles (HMPs) have recently demonstrated strong potential as universal 3D printing bioink platforms (*11*, *12*). When packed, HMPs touch and physically trap each other rather than being a free suspension in solution, resulting in having similar physical properties to bulk hydrogels (*13*). However, the interparticle interactions are much weaker compared to the covalent bonding within the HMPs, and thus, they can still yield to flow when external forces overcome the interparticle friction during printing. After HMP extrusion, the physical interactions between HMPs reestablish and can support the final structure, which confers shear-thinning properties to the jammed HMPs. HMPs remain intact during the entire process because of the stable inner-particle covalent network, protecting encapsulated cells from high shear stress and further enhancing printing stability. Their shear-thinning property is independent from the polymers and chemistries used to construct the HMPs and has been verified in a wide range of material formulations, including silica (*14*), hyaluronic acid (*11*), agarose (*11*), poly(ethylene glycol) (PEG) (*12*), chitosan (*15*), gelatin (*16*), and 2-acrylamido-2-methylpropane sulfonic acid (*17*, *18*). Printed HMP constructs can be further annealed via various secondary cross-linking strategies to improve their mechanical properties and stretchability (*12*, *16*–*18*).

**Fig. 1.**
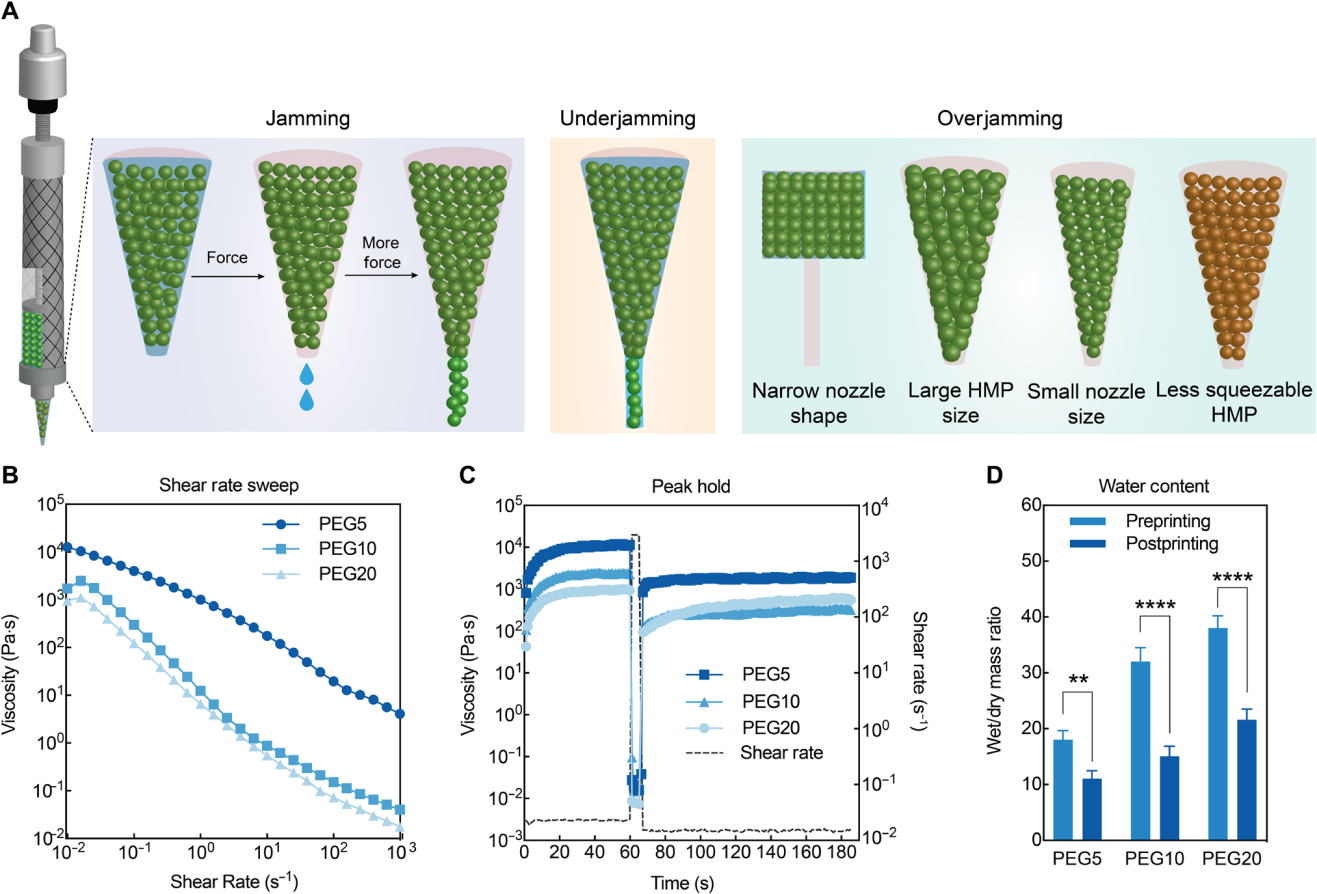
The HMP flow initiates after breakage of interparticle interactions and closer packing in syringes. (**A**) Schematic of the HMP extrusion process under varying conditions. Jamming: Interstitial water was extruded first, and HMPs were packed closer before yielding to flow. Underjamming: Due to less resistance, HMPs were extruded with interstitial water in these scenarios. Overjamming: Due to more resistance, HMPs were not extruded until rupture of the beads. (**B**) Shear rate sweeps for HMP bioinks with three different moduli, illustrating their shear-thinning characteristics. (**C**) Peak hold tests showing rapid viscosity recovery and the reestablishment of interparticle interactions in HMP bioinks, corresponding to printing performance. (**D**) Water content measurements of HMP bioinks before and after extrusion showing water loss due to the jamming process in the syringes. Student’s *t* test, **P < 0.01 and ****P < 0.0001.

Despite having the potential to be generalized for 3D printing, HMP flow during 3D printing is largely different from the liquid flow of common continuous bioinks. Two substantial underexplored aspects need to be considered for HMP bioinks. First, when HMP bioinks are loaded into syringes for printing, the wall of the syringe and nozzle provide resistance and confine the HMP flow because of the considerable size of HMPs. Depending on the size of the nozzle opening, HMPs may undergo closer packing to remove the aqueous solutions in the interstitial spaces, further deforming, squeezing, or sometimes rupturing themselves before yielding to flow (Fig. 1A). Second, the physicochemical properties of HMPs, such as size and modulus, can influence the dissipation process between HMPs. To initiate HMP flow, the external shear stress needs to surpass both the wall resistance and HMP yield stress. Therefore, these two factors are closely related to the flow initiation process for 3D printing and could potentially affect the printing quality and cytocompatibility. In this work, we elucidate how the properties of HMPs and the printing apparatus affect the flow of HMP bioinks through both experimental and computational methods. We also demonstrate how the HMP flow-initiating process influences printability and viability of encapsulated cells. These findings can be used to select printing parameters for a given HMP bioink. They also enhance the capability of HMP bioinks to print intricate 3D complex structures and can contribute to the broader use of HMP bioinks to address the need for 3D printing of engineered tissues.

## RESULTS

### Initiation of HMP flow for 3D bioprinting

We used thiol-ene PEG HMPs as a representative bioink to elucidate the mechanism underlying HMP flow for 3D printing. PEG hydrogels are common synthetic biomaterials that are difficult to print owing to their low viscosity before cross-linking, similar to most other soft biomaterials. Similar to other reported HMP bioinks (*11*, *12*, *15*), the PEG HMP pellets that jammed in the printing nozzle demonstrated shear-thinning properties under all three different moduli, indicating that interparticle frictions are dissipated under pressure to allow the movement of HMPs (Fig. 1, B and C). However, such rheological measurements ignore the wall resistance from the syringe and nozzle when HMP bioinks are printed through the syringe. Because of their particulate nature, HMPs experience higher resistance to flow than continuous liquids. The water content of HMP bioinks was reduced by almost half after being printed, regardless of the HMP cross-linking density (Fig. 1D). This result agrees with our observation that water is extruded before HMP filaments, which is attributed to the excess water in the interstitial spaces between HMPs, indicating that the physical confinement from the syringe and nozzle causes closer packing of HMPs and removes the interstitial water. The amount of interstitial water correlates to the initial water content and packing density of the HMP slurry. In our experiments, we controlled the HMP dryness by centrifuging at the same 4400 rpm speed. After jamming, HMPs can possibly undergo deformation or squeezing. The granular flow only initiates after HMPs are packed closely and the built-up forces exceed the wall resistance. Therefore, wall resistance from the printing apparatus and HMP properties can influence the packing density and dissipation of HMP bioinks and, thus, influence the final printing outcomes.

### Continuous HMP flow requires large opening of printing apparatus

**Fig. 2.**
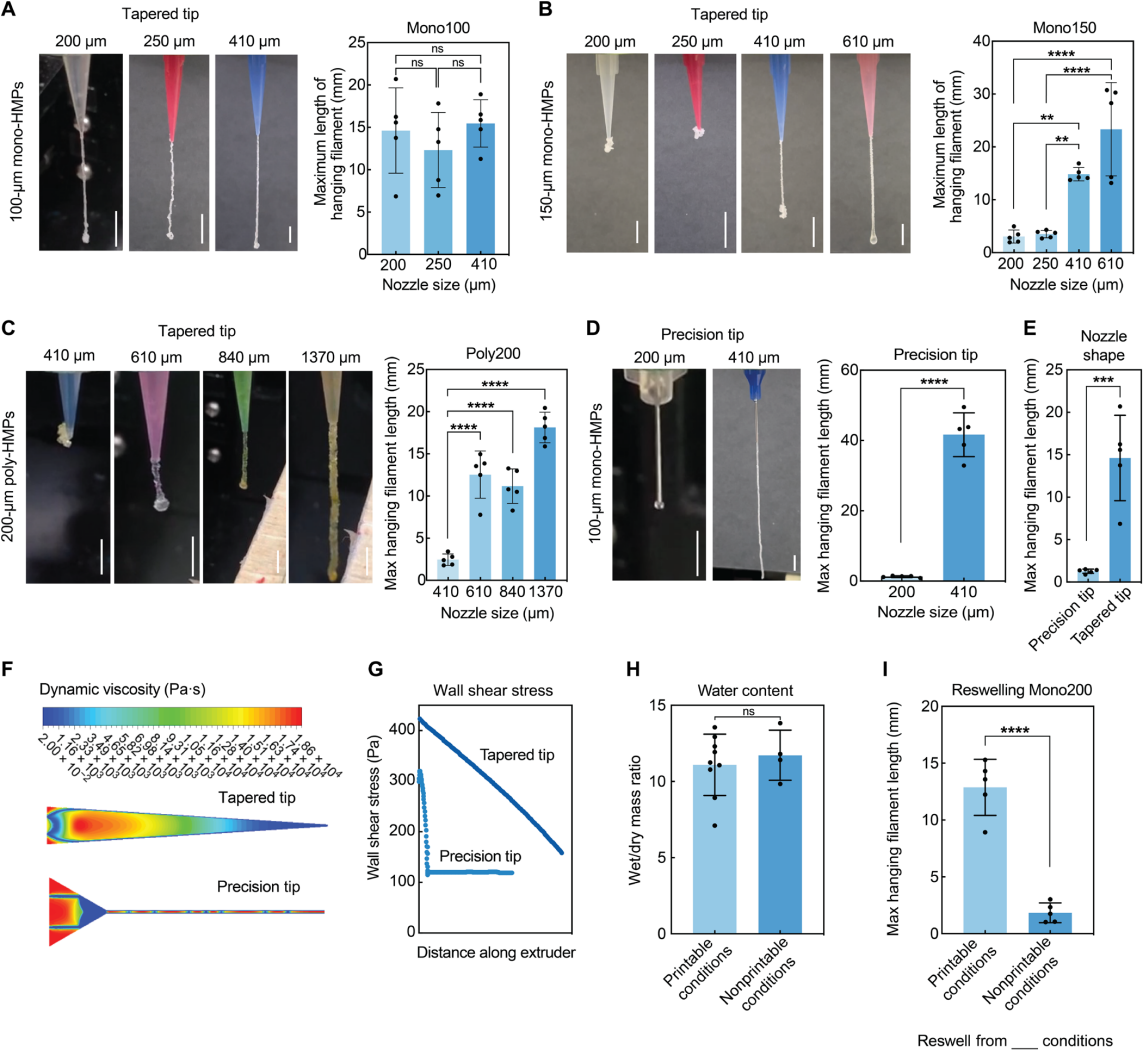
Continuous extrusion of HMP bioinks requires large opening in the syringe and low resistance. Filament extrusion tests of PEG5 HMPs compare continuity and smoothness of (**A**) 100-μm mono-HMPs in tapered tips, (**B**) 150-μm mono-HMPs in tapered tips, (**C**) 200-μm poly-HMPs in tapered tips, and (**D**) 100-μm mono-HMPs in precision tips. Varying nozzle sizes were used, and maximum length of hanging filament was measured before dropping to define printability. (**E**) Measurements of hanging filament length compared the Mono-100 HMP printability in two tip shapes. (**F**) Computational modeling of dynamic viscosity compares the extent of HMP jamming in different regions within precision tips and tapered tips. (**G**) Quantification of wall shear stress from computational modeling results in two tip shapes. (**H**) Water content measurements of PEG5 HMP bioinks after extrusion in defined printable and non-printable conditions. (**I**) Filament extrusion tests of reswelled previously extruded HMPs from both printable and nonprintable conditions in a printable condition demonstrate the damage of cross-linking network within HMPs in non-printable conditions. Scale bars, 5 mm. (A to C) One-way ANOVA with Tukey post hoc test and (D, E, G, and I) Student’s *t* test, **P < 0.01, ***P < 0.001, and ****P < 0.0001. ns, not significant. Photo Credit: Shangjing Xin & Kaivalya Deo, Texas A&M University.

To study the impact of wall resistance on HMP flow, we performed filament extrusion tests with three HMP sizes and two nozzle shapes (tapered and precision) with several nozzle diameters (Fig. 2, A to E). Generation of monodispersed HMPs of two different sizes (100 and 150 μm) was achieved by using a microfluidic gel droplet generator using varying microchannel dimensions, and polydisperse HMPs (~200 μm) were prepared via a submerged electrospraying method (figs. S1 and S2). PEG HMPs used in these experiments were photopolymerized via thiol-ene click chemistry, using 5-kDa PEG-norbornene, PEG-dithiol, Arg-Gly-Asp-Ser (RGDS) peptide, and photoinitiator. Maximum length of the hanging filament was measured under each condition to evaluate the extrudability. Under printable conditions, HMPs were extruded continuously and smoothly and formed a relatively long filament (>10 mm) before dropping, although the length of the filaments also depends on interparticle adhesion and gravity forces (movie S1). Under nonprintable conditions, HMPs were stuck in the nozzle head and burst extruded after high-pressure buildup, and long filaments were not formed (typically less than 5 mm) (movie S2). A droplet of HMPs can be seen at the end of the filament in some images, which is caused by curling of the filaments at the beginning and does not affect the printability. This filament curling will be prevented during 3D printing as extruded HMPs will touch the printing stage or the previous printing layer immediately. Our results in Fig. 2 show that a large enough opening is required to permit continuous HMP flow for printing, which can be achieved by maintaining an appropriate nozzle-to-HMP diameter ratio. For example, using tapered tips, monodisperse HMPs [Coefficient of Variation (C.V.) = 4%] were able to print when the nozzle size was more than double of the HMP size for both 100- and 150-μm HMPs, although the printable nozzle size boundary was unknown for 100-μm HMPs (Fig. 2, A and B). However, polydisperse HMPs (C.V. = 50%) required an extrusion nozzle at least three times of the average HMP size (Fig. 2C), which is attributed to the small fraction of large HMPs in polydisperse HMP batches that provides additional resistance and requiring a larger opening.

We next explored the effects of nozzle and syringe shapes on the HMP flow. For monodisperse 100-μm HMPs, using precision tips required a larger nozzle size than using tapered tips for smooth printing (Fig. 2, A, D, and E). Since the opening narrowed gradually in tapered tips, the wall resistance was distributed equally throughout the tip, whereas there was a sudden change of shape in the precision tips, which was associated with high local resistance. This was verified by mapping the dynamic viscosity and shear stress within the tips by computational fluid dynamics (CFD) simulations using Fluent Ansys v20R1 (Fig. 2, F and G). The computational modeling corroborated the experimental results and showed that the viscosity and shear stress were high at the neck of the needle within the precision tips where HMPs could be stuck. We also modeled HMP flow in 3-, 10-, and 20-ml syringes (fig. S3). These three syringes have different diameters, and thus, the resistance of HMPs merging from the syringe into the nozzle varies. Our results indicate that a 3-ml syringe is a better option for HMP printing compared to 10- or 20-ml syringes because the diameter changes in the head of the 10- or 20-ml syringes are too drastic, and the HMP bioinks need to overcome a high shear stress to flow through.

While HMPs encountered higher resistance and stress under nonprintable conditions, the water content after printing did not decrease significantly compared to the printable conditions (Fig. 2H). This result indicates that after removing the interstitial water for close packing of HMPs, the high stress did not further squeeze HMPs significantly under the nonprintable conditions. After printing, we reswelled the HMP bioinks collected from both the printable and nonprintable conditions and performed another filament extrusion test under a printable condition (Fig. 2I). Unexpectedly, only HMPs reswollen from the printable conditions were successfully printed, whereas the HMPs reswollen from the nonprintable conditions failed to jam within the nozzle, suggesting the rupture of cross-linking network within the HMPs and decreased yield strength due to the high stress under nonprintable conditions. The HMP rupture was further confirmed by confocal imaging of extruded HMPs from nonprintable conditions (fig. S4), which shows that some beads broke in half and most of the beads had rough surfaces, possibly due to tearing during extrusion. This indicates that HMP dissipation under nonprintable conditions is not sufficient to initiate HMP flow and that burst extrusion occurs after rupture of some HMPs. Collectively, these results indicate that a printing apparatus with a large opening and evenly distributed resistance is critical for smooth HMP printing.

### High printing fidelity requires refined HMPs and nozzles

**Fig. 3.**
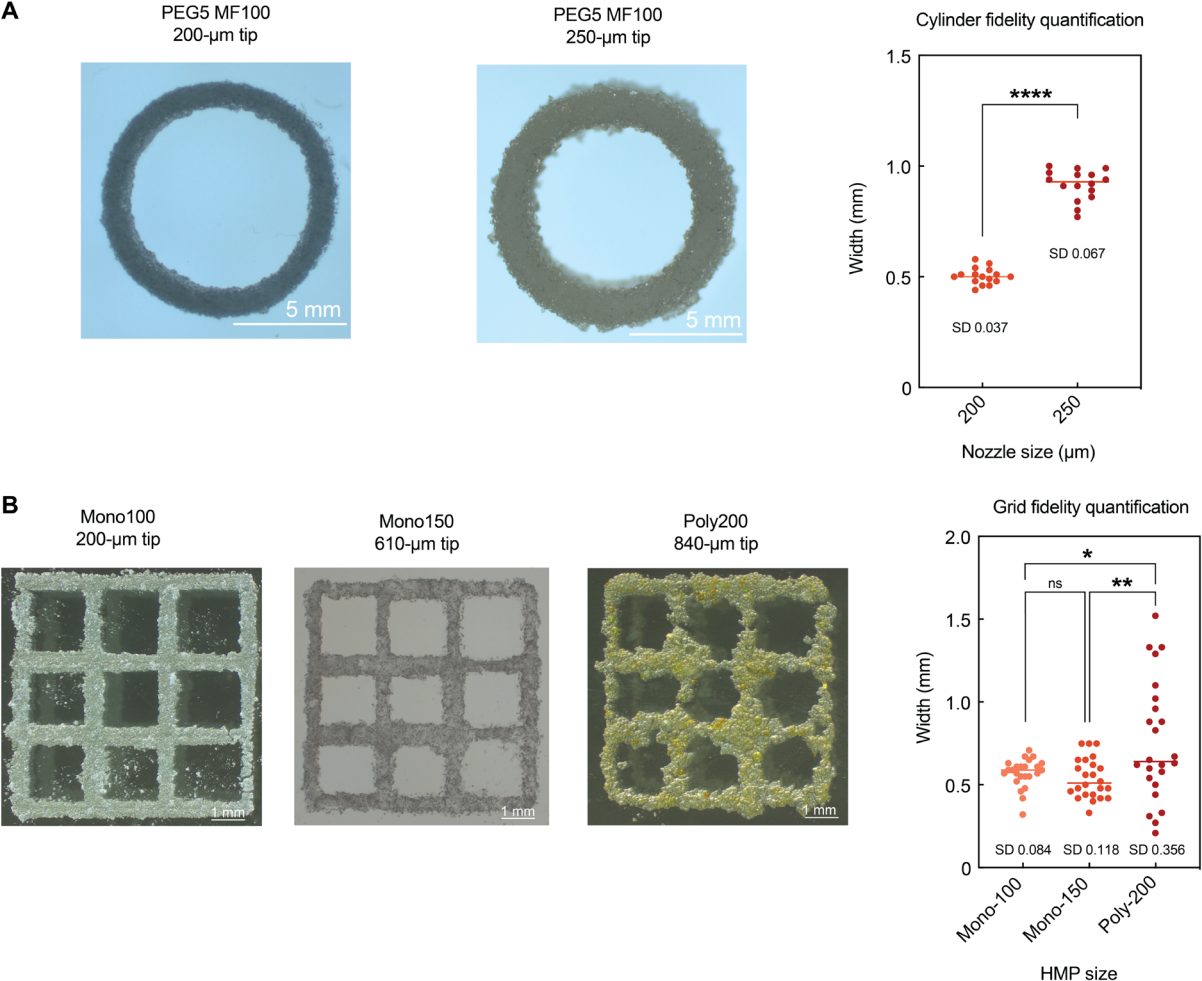
Refined HMP filaments are achieved by using smaller nozzles. (**A**) Printing of cylinder shapes using 100-μm mono-HMPs via 200- and 250-μm tapered tips to compare the printing fidelity with different nozzle sizes. (**B**) Printing of grid shapes using 100-μm mono-HMPs, 150-μm mono-HMPs, and 200-μm poly-HMPs in their optimized conditions further reveals that more precise and refined printing requires smaller HMPs and nozzles. (A) Student’s *t* test and (B) one-way ANOVA with Tukey post hoc test, *P < 0.05, **P < 0.01, and ****P < 0.0001. Photo Credit: Shangjing Xin & Kaivalya Deo, Texas A&M University.

We next studied the effects of the printing conditions on fidelity (Fig. 3). While HMPs can generate consistent filaments as long as the nozzle provides a large enough opening, the width of these filaments correlates to the size of the nozzle directly. Because of the particulate nature of HMP bioinks, they cannot be stretched as other continuous bioinks after extrusion. Thus, it is difficult to print thin filaments using a large nozzle for HMP bioinks simply by adjusting the printing speed. Therefore, the minimal nozzle size should be used to achieve the best printing fidelity. When printing 100-μm HMPs, using a 200-μm tip can result in a printed ring structure with significantly thinner filaments compared to using a 250-μm tip (Fig. 3A). Therefore, it is important to pick a nozzle with the smallest extrudable diameter to achieve a balance between extrudability and fidelity. In addition, HMPs with different sizes and polydispersity were used to print a grid shape using their smallest printable nozzle size (Fig. 3B). Here, the polydisperse HMPs could not produce filaments with consistent width. While the width measurements between the two different size monodisperse HMP-printed grids showed no significant difference, 100-μm HMPs resulted in much more consistent filaments (lower SD as labeled in Fig. 3B) and cleaner intersections between gridlines compared to 150-μm HMPs, suggesting that smaller monodisperse HMPs and nozzles are beneficial for improving printing fidelity.

### The extent of HMP jamming influences printing flow and stability

**Fig. 4.**
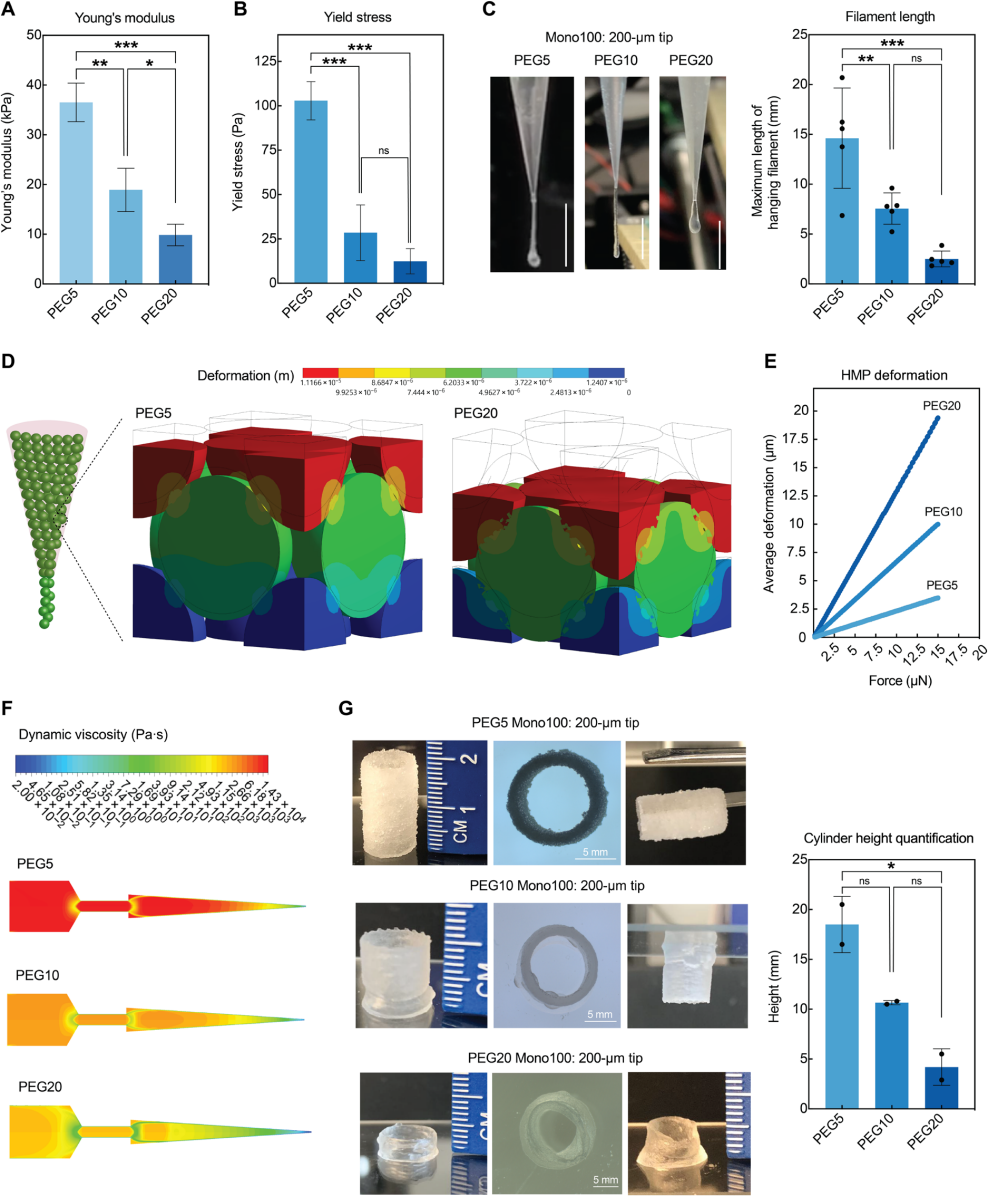
Mechanical properties of HMP bioinks influence their dissipation, deformation, and printing stability. (**A**) Atomic force microscopy nanoindentation measurements of Young’s modulus of PEG5, PEG10, and PEG20 HMPs. (**B**) Yield stress from strain sweeps for PEG5, PEG10, and PEG20 HMPs showing the forces required to dissipate the HMPs. (**C**) Filament extrusion tests for PEG5, PEG10, and PEG20 HMPs via 200-μm tapered tips and quantification of hanging filament length. Scale bars, 5 mm. (**D**) Image showing the computational modeling on HMP deformation in a closed FCC cubic with gradually increasing force up to 15 μN applied from the top. (**E**) Quantification of displacement distance during deformation under the gradually increasing force for PEG5, PEG10, and PEG20 HMPs. (**F**) Computational modeling of dynamic viscosity comparing the extent of HMP jamming during 3D printing as a function of modulus. (**G**) Printing of cylinder shape to the maximum height before collapse demonstrating the difference in printing stability as a result of HMP modulus and extent of jamming. One-way ANOVA with Tukey post hoc test, *P < 0.05, **P < 0.01, and ***P < 0.001. Photo credit: Shangjing Xin and Kaivalya Deo, Texas A&M University.

We next explored the effects of HMP mechanical properties on printability. We used three molecular weights (MWs) of PEG-norbornene to fabricate HMPs with similar postswelling size (80 to 100 μm), and they exhibited varying moduli due to the difference in cross-linking density (Fig. 4A). On the basis of the rheological strain sweep curves, PEG5 had a higher yield stress compared to PEG10 and PEG20, which indicated that the interparticle interactions were stronger between PEG5 HMPs, possibly due to more polymer chain penetration into the surrounding HMPs as a result of higher polymer density (Fig. 4B and fig. S5) (*19*). In filament extrusion tests using a 200-μm tapered tip, all three HMPs formed continuous filaments, but PEG5 HMPs generated longer hanging filaments (Fig. 4C), which was attributed to the higher interparticle adhesion. In addition, HMP deformation was tested by simulating the deformation of a closed face-centered cubic (FCC) packing of the HMPs under compressive load using the Ansys Static Structural software (Ansys v20R1) (Fig. 4D and fig. S6). FCC packing was chosen as it has the highest average packing density and thus mimics the close HMP packing after jamming in the printing nozzle before initiation of HMP flow. A force of 15 μN from the top was modeled as larger forces tend to affect the convergence of the static solution. When applying the same force, closely packed PEG20 HMPs deformed more compared to PEG5 HMPs (Fig. 4E). Since PEG20 HMPs were more subject to deformation, they would experience less stress and resistance flowing through the nozzle compared to PEG5 HMPs, even with the same HMP size and nozzle opening. Again, we mapped the viscosity and wall shear stress throughout the syringe and nozzle by CFD simulations (Fig. 4F and figs. S7 to S9). The results further revealed that the overall resistance was significantly lower when printing PEG20 HMPs compared to PEG5 HMPs.

These HMP bioinks with varying stiffness were then printed into 10-mm-diameter cylinders until the maximum heights were reached before collapsing (Fig. 4G). Because all printed cylinders were evaluated without secondary cross-linking, this experiment tested the strength of interparticle adhesion as a function of HMP stiffness by quantifying the *z*-axis stability of the printed cylinders. PEG5 HMPs successfully printed cylinders up to 2 cm in height, whereas PEG20 HMPs collapsed after reaching a height of 5 mm. We also demonstrate that the PEG5 cylinder could be lifted by forceps without falling, further indicating the strong interparticle adhesion. The differences in the interactions between HMPs were mainly attributed to the process of initiating the HMP flow during printing. As PEG5 HMPs experienced higher stress and resistance, they were packed closer in the syringe before extrusion, and thus, more polymer chains penetrated into the surrounding HMPs. Therefore, the jamming and dissipation process is crucial to the quality of the final printed products.

### The extent of HMP jamming influences cell viability

**Fig. 5.**
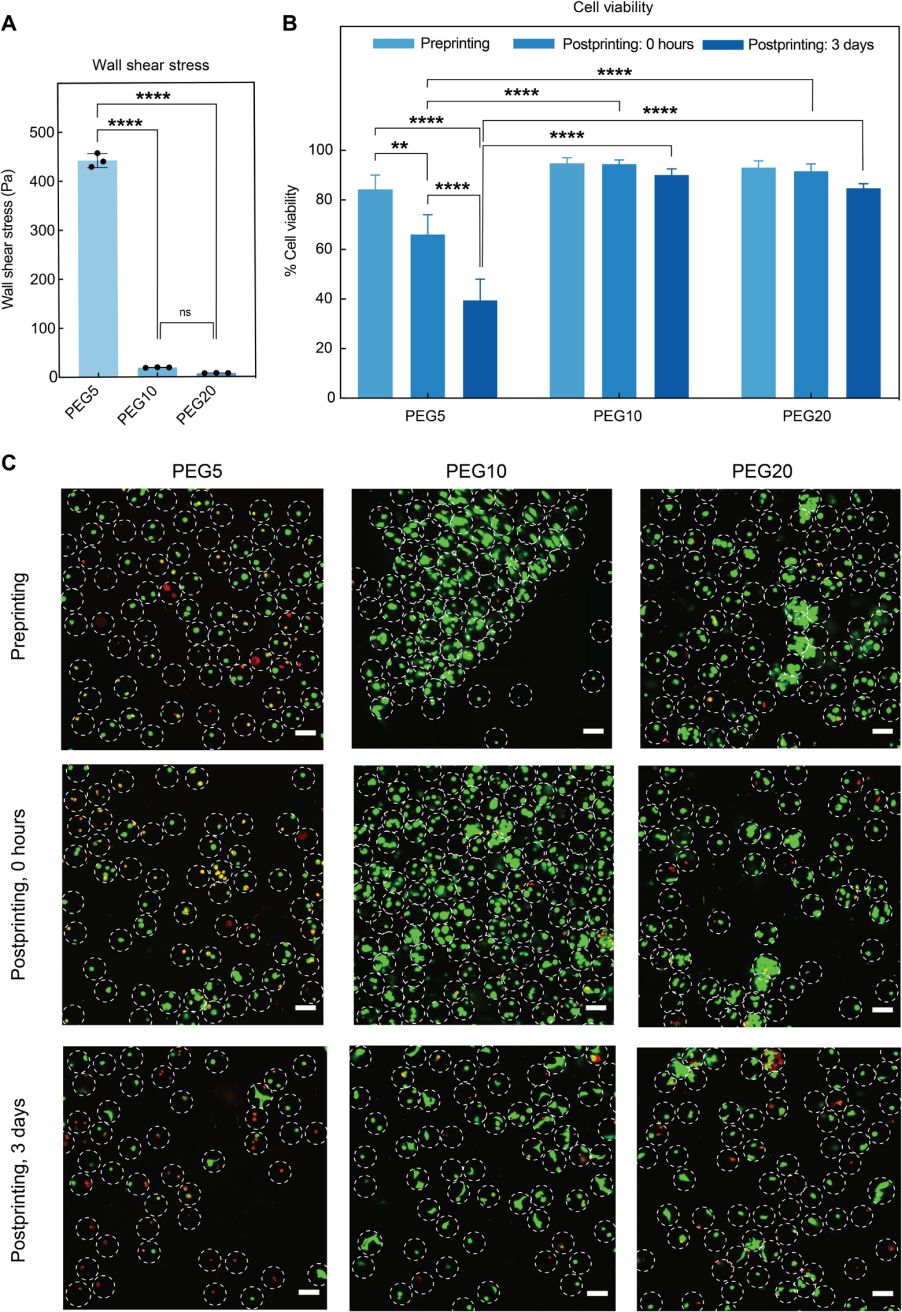
Mechanical properties of HMPs influence viability of encapsulated cells as stress relates to the extent of jamming. (**A**) Maximum wall shear stress from dynamic viscosity computational results when extruding PEG5, PEG10, and PEG20 HMPs. (**B** and **C**) Z-projection confocal images and quantification of live/dead staining of hMSCs encapsulated within HMPs before and after printing. (A) One-way ANOVA and (B) two-way ANOVA with Tukey post hoc test, **P < 0.01 and ****P < 0.0001.

We also examined whether the difference in HMP jamming before yielding to flow influences biocompatibility of the cells encapsulated within the HMPs (Fig. 5). Human mesenchymal stem cells (hMSCs) were encapsulated within the 100-μm HMPs using a microfluidic gel droplet generator, and the produced cell-laden HMPs were printed through a 200-μm tapered tip immediately after washing off the oil phase. Since PEG5 HMPs were more resistant to deform, they experienced significantly higher wall shear stress compared to PEG10 and PEG20 HMPs based on the data through computational modeling (Fig. 5A). The encapsulated hMSCs exhibited high cell viability after microfluidic encapsulation in all three stiffness groups. However, about 40% of cells died immediately after extrusion within the PEG5 HMPs, whereas the cell viabilities were above 90% in both the PEG10 and PEG20 HMPs. The 20% decrease in cell viability in the 0-hour PEG5 HMPs postprinting compared to preprinting suggests that stiff HMPs impair cell viability due to the high stress they generate during extrusion. After 3 days of culture, the cell viability continued to decrease in the PEG5 HMPs, possibly due to both the damage during the extrusion process and the restrictive environment of the PEG5 cross-linked network (*20*, *21*). This result indicates that there is a balance between achieving excellent printing stability and maintaining high cell viability when selecting the optimum HMP stiffness. Therefore, the extent of HMP jamming needs to be controlled carefully, where our data indicated that PEG10 HMPs can achieve both high printing stability and high cytocompatibility at the same time.

## DISCUSSION

Many biomaterial formulations cannot be leveraged for 3D bioprinting to fabricate intricate biofunctional structures for tissue engineering applications because of their poor printability (*22*). Recent advances suggest that the printability of these soft biomaterials can be largely improved after cross-linking them into solid granules or HMPs (*23*). These HMP-based bioinks have shear-thinning characteristics and are printable because the weak interparticle interactions can be broken upon external forces to enable the movement of HMPs. However, the process of HMP jamming and dissipation within the 3D printing apparatus remains unclear and, thus, represents a knowledge gap to further improve the printability of HMP bioinks. Here, we elucidated the impacts of (i) external resistance from varying shapes and openings of the printing apparatus and (ii) internal mechanical properties and sizes of HMPs on viscous dissipation and flow initiation.

Our experimental and computational results revealed that the wall shear resistance from syringes and nozzles to the HMP bioinks could be greater than the interparticle frictions, meaning HMPs do not yield similarly as under rheological measurements. Instead, they packed further within the syringes until the built-up forces surpassed the wall resistance. Therefore, a large enough opening was required for smooth printing of HMP bioinks, but the shape and size of the syringe and nozzle as well as the size and polydispersity of the HMPs must also be considered. A small opening caused rupture of HMPs before yielding to flow. However, high printing fidelity required a small opening of the nozzle. Therefore, the choice of syringe and nozzle needs to consider the trade-off between extrudability and fidelity. In addition, softer HMPs were more subject to deformation and could squeeze through tight nozzles. Thus, they required a smaller opening compared to stiffer HMPs.

The jamming process within the syringes also affected the printing stability and cytocompatibility. If the HMPs were jammed to a greater extent, the interparticle adhesion was enhanced possibly due to penetration of dangling polymer chains between HMPs and resulted in excellent stability without requiring secondary cross-linking. This is particularly important for HMP bioprinting as the interparticle adhesion allows printing intricate structures, such as overhangs, without the need for supporting baths. Printed structures can be easily handled for secondary cross-linking after the printing process is completed, and the interparticle adhesion is maintained unless the printed constructs are submerged into solutions. The interparticle adhesion and potential secondary cross-linking decrease the porosity between HMPs, which helps to achieve complete filling within the designed shape, as required by many 3D printing applications. If porosity in printed constructs is required, some sacrificial microparticles can be mixed in the bioink and removed after printing to create pores (*24*). In addition, our results also demonstrated that the overjamming of HMPs in the syringe resulted in high stress and led to reduced cell viability after printing. Together, our PEG10 formulation achieved a balance of excellent cytocompatibility and printing stability at the same time. When taking these factors into consideration, the printability of HMP bioinks can be improved to expand their applications. Furthermore, since HMPs have also recently been used as injectable biomaterials to construct porous scaffolds for tissue repair (*25*, *26*), these findings provide information on how to select appropriate syringes and needles for in vivo injection. Since HMPs are injected into tissue cavities in these applications, the stability after injection becomes less important, but it is crucial to maintain a steady and consistent flow to precisely control the injection volume and avoid damage to surrounding soft tissues. Therefore, minimizing the jamming and maintaining a large opening would be important for in vivo injections of HMPs.

While we believe that our conclusions on the jamming process and initiation of HMP flow can be generalized to HMP bioinks made from many different biomaterial formulations, the optimized values reported here, such as HMP/nozzle size ratios and HMP stiffness, may vary depending on the polymer type and cross-linking chemistry. In addition, the properties of dangling chemical chains on HMPs depend on polymer chemistry, such as charge, and can result in varying bonding strength between HMPs and printing stability, which needs further investigation. Our computational models on dynamic viscosity mapping only consider the HMP modulus and lack consideration of the particulate nature, such as size and packing density. Furthermore, the smallest nozzle we used was 200 μm in size because of the limitations of commercial availability. Future studies can engineer smaller nozzles to print smaller HMPs for more confined structures, as current HMP-based 3D bioprinting was limited to coarse filaments owing to the relatively large nozzle size. We envision the potential use of a 40-μm tapered tip to print 10- to 20-μm monodisperse HMPs to produce more intricate structures in native tissues, or beads around 50 μm may be used for printing cell-laden HMPs with a higher printing resolution. The jamming details of granular hydrogels in shapes other than spheres should also be studied, as, for example, entangled microstrands have been used in 3D bioprinting recently (*27*), and the mechanism of flow yielding could be different. Last, future work can leverage the indiffusivity of HMP bioinks (*28*) to 3D print heterogeneous biological structures (*29*), which otherwise require complicated setups for continuous bioinks (*30*).

## MATERIALS AND METHODS

### Fabrication of microfluidic HMPs

The microfluidic device was made of polydimethylsiloxane (PDMS) by standard soft lithography. Master molds were fabricated on a 4-inch silicon wafer by a photolithographic technique using a negative photoresistor (SU8 2075, MicoChem). Microfluidic devices were molded from master molds by pouring degassed PDMS (Sylgard 184, Dow, elastomer:cross-linker = 10:1) and cured at 85°C for 1 hour. PDMS devices were then placed onto a glass slide coated with PDMS (elastomer:cross-linker = 20:1) and bonded together at 85°C overnight. HMP droplets were generated at the flow focusing region where the oil phase broke off the gel solution into droplets (fig. S1). The gel solutions consisted of 10 weight % (wt %) four-arm PEG-norbornene [synthesized from four-arm PEG-hydroxyl as previously reported (*31*); JenKem Technology; MW = 5, 10, and 20 kDa, termed PEG5, PEG10, and PEG20 groups, respectively], PEG-dithiol (Laysan Bio; MW = 3400 Da, resulting [SH]:[ene] ratio of 0.75:1), lithium phenyl-2,4,6-trimethylbenzoylphosphinate [6 mM; synthesized as previously described (*32*)], and Cys-Gly-Arg-Gly-Asp-Ser (CGRGDS) (1 mM; synthesized via 9-fluorenylmethyloxycarbonyl solid-phase peptide synthesis). For cell encapsulation, PEG-dithiol was replaced with matrix metalloproteinase–degradable peptide cross-linker Lys-Cys-Gly-Pro-Gln-Gly-Ile-Trp-Gly-Gln-Cys-Lys (KCGPQGIWGQCK) (AAPPtec) at the same [SH]:[ene] ratio. The oil phase included light mineral oil with Span 80 (5 wt %). The HMP droplets were cured downstream with ultraviolet (UV) irradiation (20 mW cm^−2^, 365 nm).

Because of the differences in swelling ratios, HMPs were fabricated with the goal of having the same 100-μm postswelling size for PEG5, PEG10, and PEG20 groups. For PEG5 100-μm mono-HMPs, the channel dimensions were 67 μm in height and 150 μm in width for the oil phase, and 120 μm in width for the gel phase. The oil phase and gel phase flow rates were 300 and 120 μl/hour, respectively. For PEG5 150-μm mono-HMPs, the channel dimensions were 117 μm in height and 200 μm in width for the oil phase, and 200 μm in width for the gel phase. The oil phase and gel phase flow rates were 500 and 200 μl/hour, respectively. For PEG10 100-μm mono-HMPs, the dimensions were 67 μm in height and 100 μm in width for the oil phase, and 80 μm in width for the gel phase. The oil phase and gel phase flow rates were 150 and 90 μl/hour, respectively. For PEG20 100-μm mono-HMPs, the channel dimensions were 51 μm in height and 30 μm in width for the oil phase, and 30 μm in width for the gel phase. The oil phase and gel phase flow rates were 60 and 20 μl/hour, respectively.

### Fabrication of electrosprayed HMPs

Polydisperse PEG5 HMPs were prepared via submerged electrospraying, as shown in fig. S1. The same precursor solutions as described above were electrosprayed into a bath of light mineral oil with Span 80 (0.5 wt %) and photopolymerized into HMPs with UV irradiation (60 mW cm^−2^, 365 nm). The UV light was kept on for 2 min after all precursor solutions were sprayed. The HMPs were rinsed with 1× phosphate-buffered saline (PBS) three times and 30% ethanol once via centrifugation at 4400 rpm for 5 min to remove the mineral oil. The HMPs were stored in PBS at 4°C and allowed to reach equilibrium swelling before use.

### Characterization of HMPs

Because of the large quantity of HMPs required for 3D printing, we combined HMPs from multiple batches, and all the characterization was performed on the pooled samples. The morphology of the HMPs was observed by a light microscope (Axio Observer Z1, Zeiss). The sizes of the HMPs were measured from images using ImageJ software. Fifty to 100 microgels per microfluidic group and 300 microgels for the electrosprayed group were quantified. HMP pellets were also cryosectioned into 25-μm slices, and the Young’s modulus of the HMPs was tested by atomic force microscopy (Dimension Icon, Bruker) with a SiO_2_ colloidal probe (5 μm diameter, spring constants of 0.6 N m^−1^; Novascan). A Discovery Hybrid Rheometer 2 (DHR-2, TA Instruments) with an attached 20-mm parallel plate at a gap height of 0.25 mm and 25°C was used for all rheological experiments. Rotational shear rate sweeps were performed between 10^−2^ and 10^3^ (s^−1^) to determine the shear-thinning behavior of the HMPs. Rotational time sweeps were executed at three different shear rates (s^−1^) in sequential order: 10^−2^ (60 s), 3000 (5 s), and 10^−2^ (120 s) to evaluate shear recovery of solutions after application of a high shear rate. The strain sweep was performed at 1 Hz in a strain range from 0.1 to 1000%. Yield stress was reported by noting the corresponding value of oscillatory stress at the crossover points of storage and loss modulus. The water content of densely packed HMPs was measured by comparing the wet/dry mass ratios of HMPs before and after extrusion via a 3D printer.

### 3D printing

Printed shapes were designed in SolidWorks and exported as STereoLithography (STL) files. The STL files were loaded into Slic3r Prusa edition 1.31.6 to customize printing options and converted into G-code printer instructions. Repetier-Host was used to interface with the 3D printer. The layer height was set to 160 μm, the layer width was set at 200 μm, and the print speed was kept at 10 mm s^−1^ or 0.32 ml min^−1^. HMP pastes were loaded into a syringe and inserted into an extrusion tube. The interstitial water was removed first until an HMP filament came out for all extrusion tests and 3D printing. HMPs were then extrusion-printed through an I3 RepRap printer. For extrusion tests, HMPs were extruded at a constant speed of 10 mm s^−1^ or 0.32 ml min^−1^. HMP bioinks were extruded five times in each condition, and the maximum length of the hanging filament in each extrusion was measured. A 6-cm square grid and a hollow cylinder with an outer diameter of 10 mm and an inner diameter of 9 mm were printed from the HMP bioink to evaluate the printing fidelity and stability in varying conditions.

### Computational modeling

#### CFD simulation of the extrusion process

The mass and momentum equations for an incompressible steady fluid flow were solved using the CFD software package Fluent (Ansys v20R1) based on a finite volume scheme. Since the 3D domain has an axis of symmetry, we reduced the dimensionality of the domain and solved the fluid flow in it as an axisymmetric problem. We considered the fluid domain to be 2D, the fluid to be incompressible, and the flow to be steady. The densely packed HMPs were modeled as a non-Newtonian fluid with constant density and viscosity based on shear rate sweep data (Fig. 1B). The rheological data were attempted to fit various models, including Power-law, Herschel-Bulkley, and Carreau, for obtaining the required shear-thinning and flow constants for simulation, and the best *R*^2^ fit for the data was obtained in the Carreau model (table S1). The Carreau model for viscosity was as follows

$$\mu =\mu_{\infty }+(\mu_{0}-\mu_{\infty }){\left(1+{\left(\lambda \overset{\cdot }{\gamma }\right)}^{2}\right)}^{\frac{\left(n-1\right)}{2}}$$

where μ, $\overset{\cdot }{\gamma }$, λ, and *n* are viscosity, shear rate, relaxation time, and flow behavior index, respectively. μ_∞_ and μ_0_ are the viscosity at infinite shear rate and zero shear rate, respectively. The inlet boundary condition for the simulation was a “velocity” corresponding to the extruder plunger velocity, and the outlet boundary condition was a “pressure” outlet condition set to an atmospheric gauge pressure of zero.

#### Structural mechanics simulation of HMP interaction

The structural interaction between the HMPs was studied using the finite element analysis software package Ansys Static Structural (Ansys v20R1). HMPs of 100 μm diameter were assumed to have a face-centered closed packing (FCC packing) under the extrusion pressure. A single lattice of the FCC packing of the beads was modeled in SolidWorks 2020 (v28) and exported to Ansys SpaceClaim (from which the model was imported to Ansys Static Structural). The HMPs were then discretized before the application of relevant boundary conditions. The HMPs were applied with symmetry boundary conditions at lattice boundaries. The contacts between the HMPs were modeled as bonded contacts. A pressure corresponding to the extrusion pressure was applied at the top of the HMPs. We analyzed the total deformation of the lattice shape for HMPs with varying stiffness under the extrusion process.

### Cell culture and encapsulation

hMSCs were acquired from the Institute of Regenerative Medicine at Texas A&M University and cultured in α-minimal essential medium (Gibco) supplemented with 20% (v/v) fetal bovine serum (Atlanta Biologicals), 2 × 10^−3^ M GlutaMAX (Gibco), penicillin (50 U ml^−1^; Gibco), and streptomycin (50 μg ml^−1^; Gibco) at 5% CO_2_ and 37°C in a humidified environment. hMSCs were used up to passage 5. After trypsinization, hMSC suspensions were mixed within 1.25× gel precursor solution in a density of 6 million cells/ml. Cell density gradient medium, OptiPrep (Sigma-Aldrich), was added to dilute the gel precursor solution to 1×, which maintained cells in suspension during the microfluidic process. A similar microfluidic droplet fabrication process was performed to make 100-μm HMPs, and UV irradiation (20 mW cm^−2^, 365 nm) was applied to a spot with a 5-cm diameter on the outlet tubing. The cell-laden HMPs were washed with 1× PBS three times via centrifugation at 1000 rpm for 5 min to remove the mineral oil. After the last wash, the supernatant was aspirated, and the HMPs were immediately loaded into syringes for 3D printing test using a 200-μm tapered tip. Postprinting HMPs were collected for cytocompatibility evaluation either immediately after printing or after 3 days of culture. They were then stained using a Live/Dead viability kit (L3224, Invitrogen) and imaged in a glass-bottom petri dish (MarTek) by confocal microscopy (FV1000, Olympus).

### Statistical analysis

All data are presented as means ± SD, and statistical analysis was performed using GraphPad Prism 9. All statistical comparisons were made using Student’s *t* test, one-way analysis of variance (ANOVA) with Tukey post hoc test, or two-way ANOVA Tukey post hoc test. Statistical significance is presented as *P < 0.05, **P < 0.01, ***P < 0.001, and ****P < 0.0001.
