# Supplementary material for: Generalizing hydrogel microparticles into a new class of bioinks for extrusion bioprinting
Source: Sci Adv. 2021 Oct 15;7(42):eabk3087. doi: 10.1126/sciadv.abk3087 (PMC8519565; doi:10.1126/sciadv.abk3087)
Supplement: Supplementary file 1 — Figs. S1 to S9 Table S1 Legends for movies S1 and S2 [file sciadv.abk3087_sm.pdf]

Science Advances
AAAS

# Supplementary Materials for

## Generalizing hydrogel microparticles into a new class of bioinks for extrusion bioprinting

Shangjing Xin, Kaivalya A. Deo, Jing Dai, Navaneeth Krishna Rajeeva Pandian, David Chimene, Robert M. Moebius, Abhishek Jain, Arum Han, Akhilesh K. Gaharwar*, Daniel L. Alge*

*Corresponding author. Email: dalge@tamu.edu (D.L.A.); gaharwar@tamu.edu (A.K.G.)

Published 15 October 2021, *Sci. Adv.* **7**, eabk3087 (2021)
DOI: 10.1126/sciadv.abk3087

**The PDF file includes:**

Figs. S1 to S9
Table S1
Legends for movies S1 and S2

**Other Supplementary Material for this manuscript includes the following:**

Movies S1 and S2

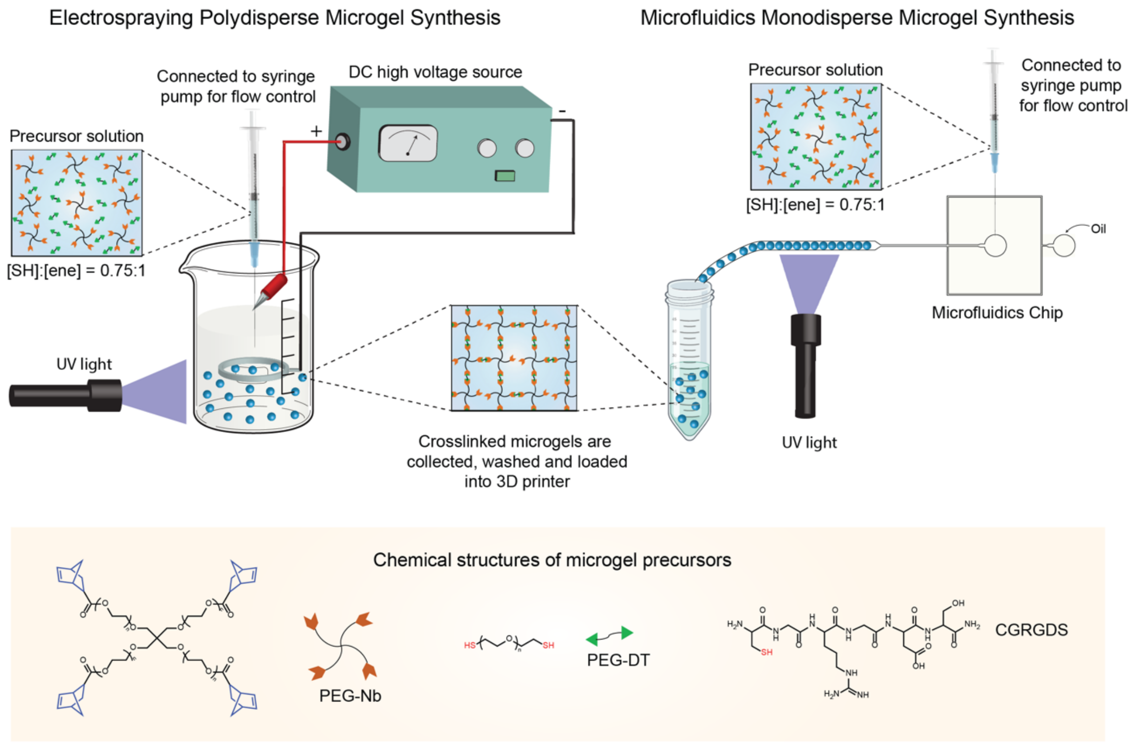


**Fig. S1.**

**Schematic of the hydrogel microparticle fabrication methods.** Droplet microfluidics and submerged electrospraying methods were used to fabricate monodisperse and polydisperse HMPs, respectively, via thiol-ene photopolymerization.

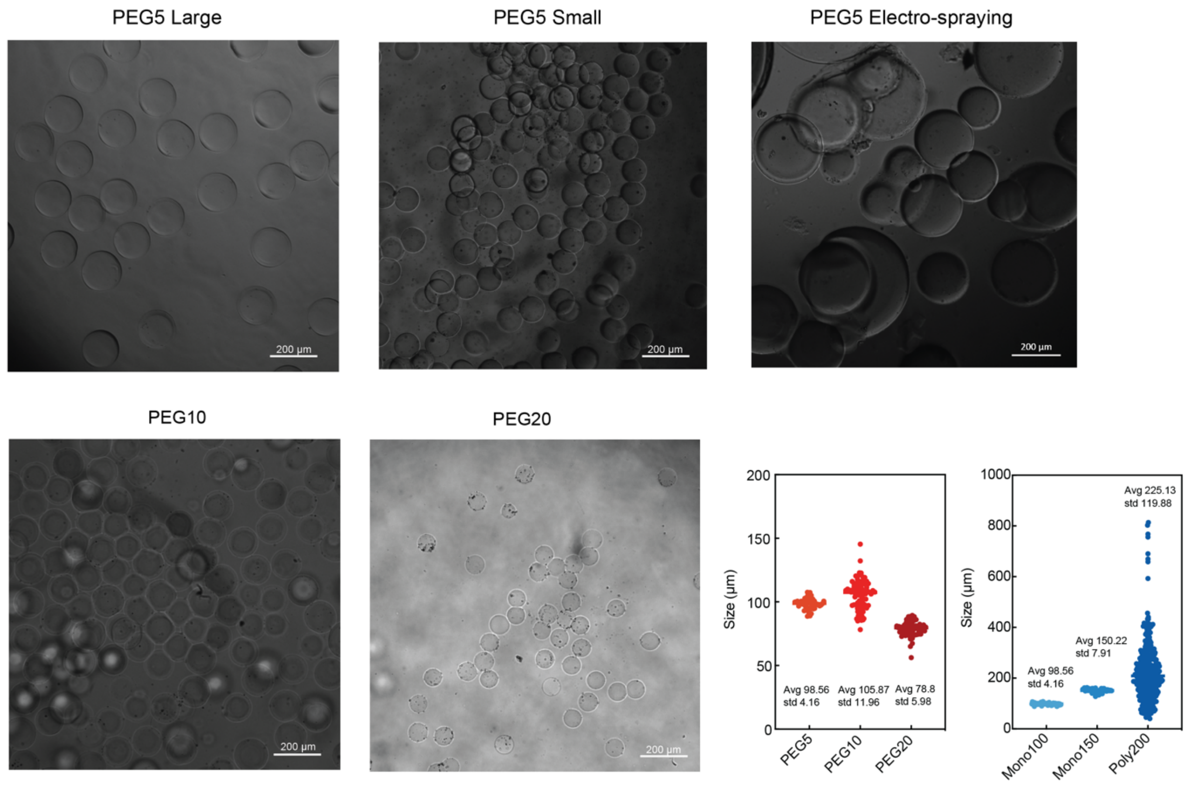


**Fig. S2.**

**Size characterization of HMPs.** Size quantification was performed using ImageJ software.

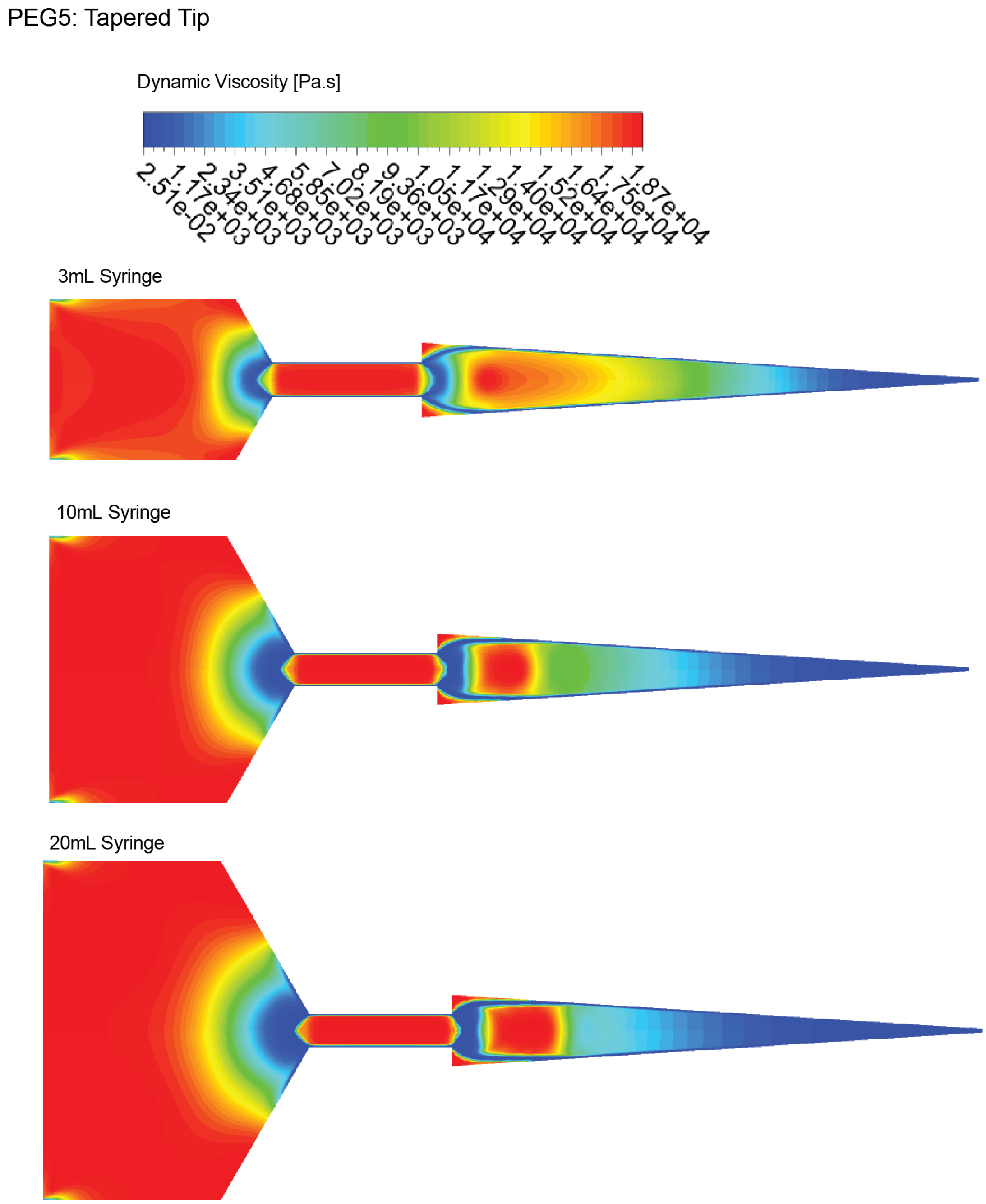


**Fig. S3.**
**Dynamic viscosity simulations of PEG5 HMP printing using 3 mL, 10 mL, and 20 mL syringes.**

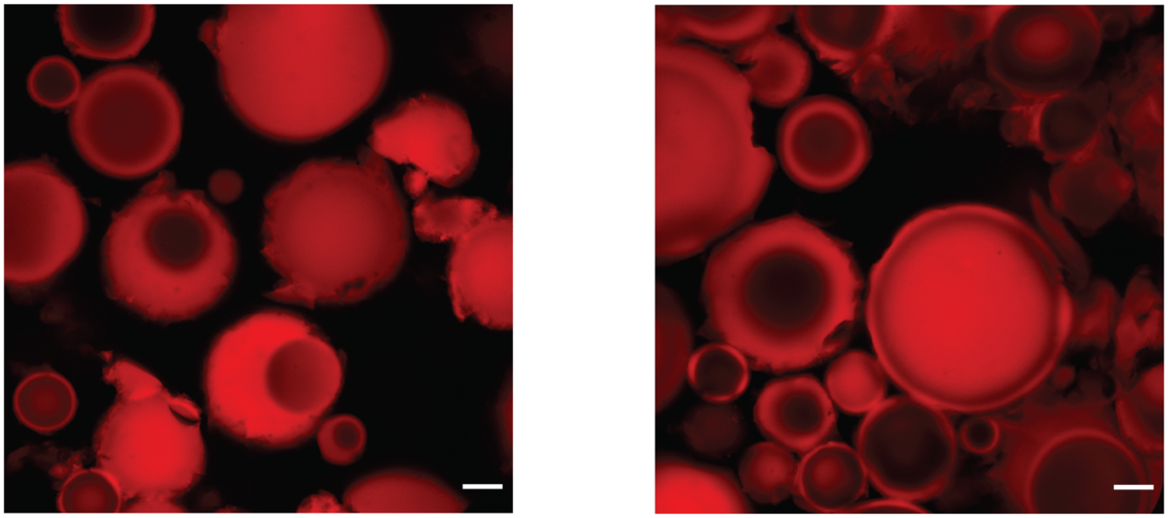

**Fig. S4.**

**Average intensity Z-projection confocal images of broken HMPs after printing through non-printable conditions.** HMPs were stained with Alexa Fluor 555-tetrazine. The scale bars are 100 µm.

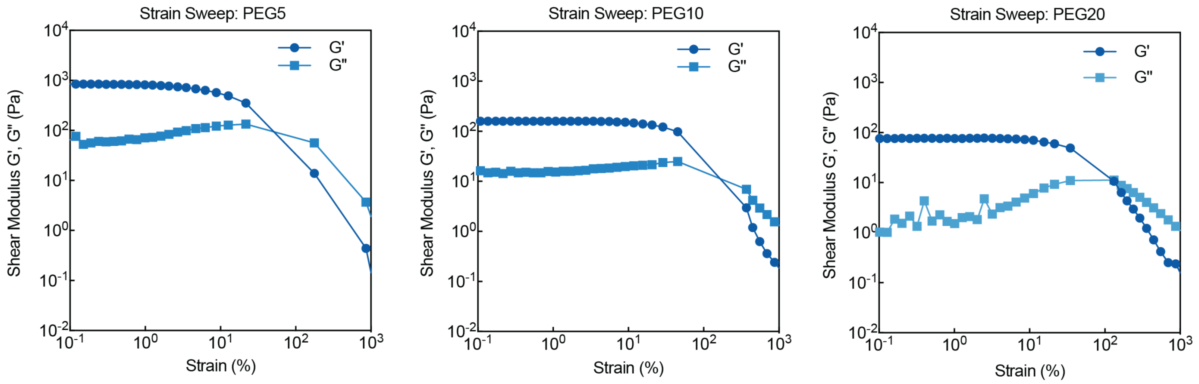


**Fig. S5.**

**Yield stress of PEG5, PEG10, and PEG20 HMPs.** Rheological strain sweeps comparing the force required for HMP yielding based on the crossover of storage and loss modulus.

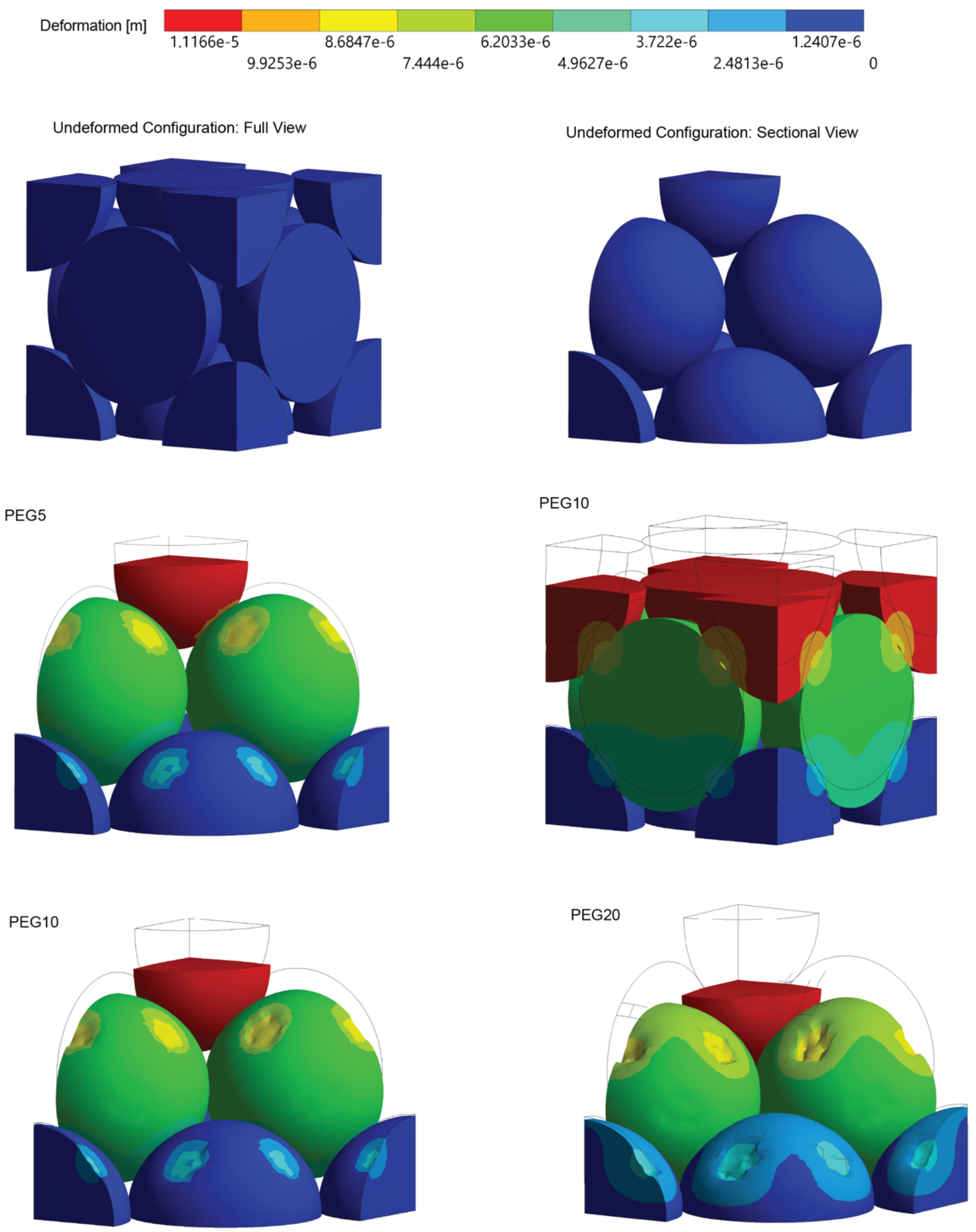


**Fig. S6.**

**Simulations on HMP deformation in closed FCC cubics with an increasing force from top up to 15 µN.**

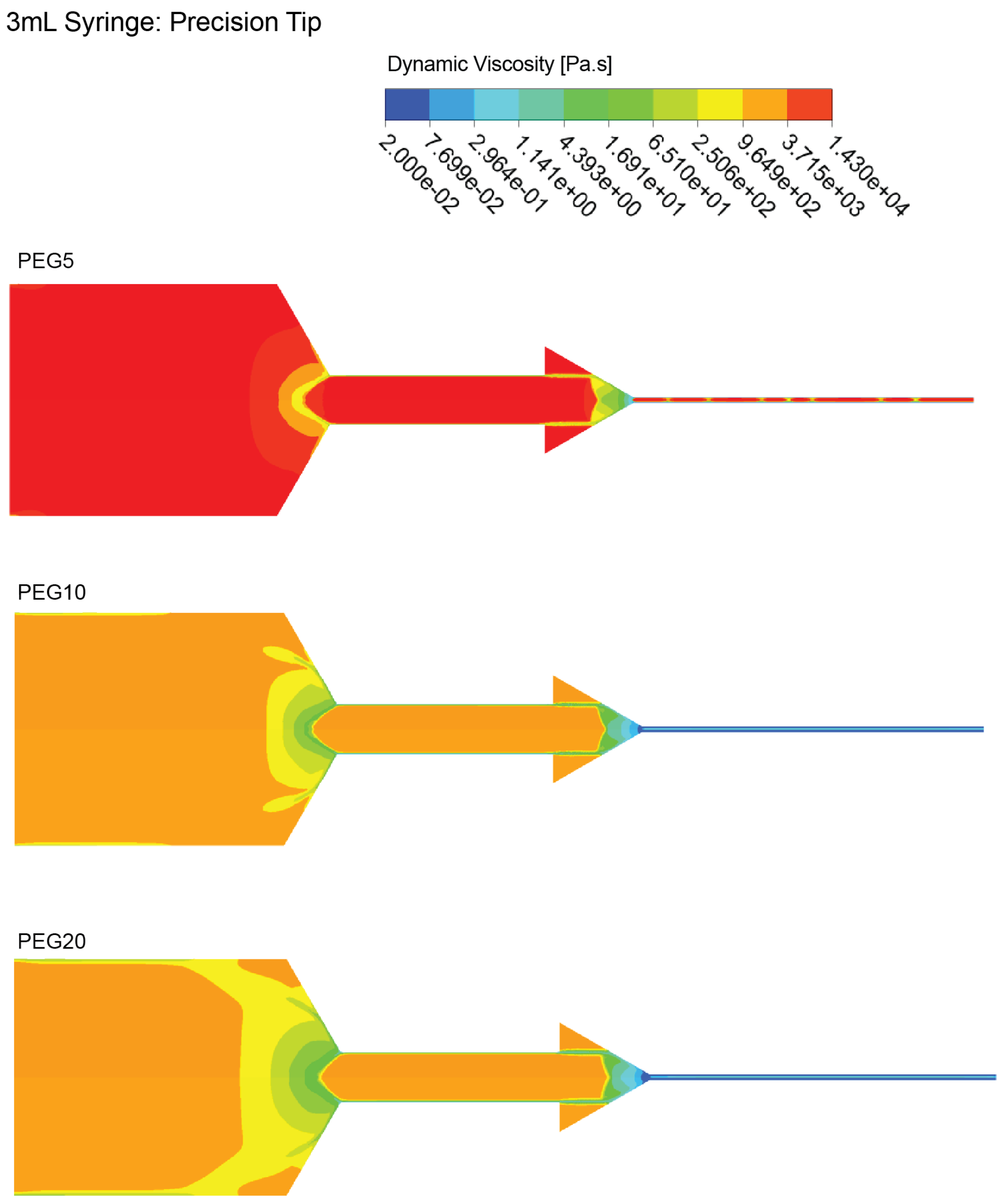


**Fig. S7.**

**Dynamic viscosity simulations of PEG5, PEG10, and PEG20 HMP printing using 3 mL syringes and precision tips.**

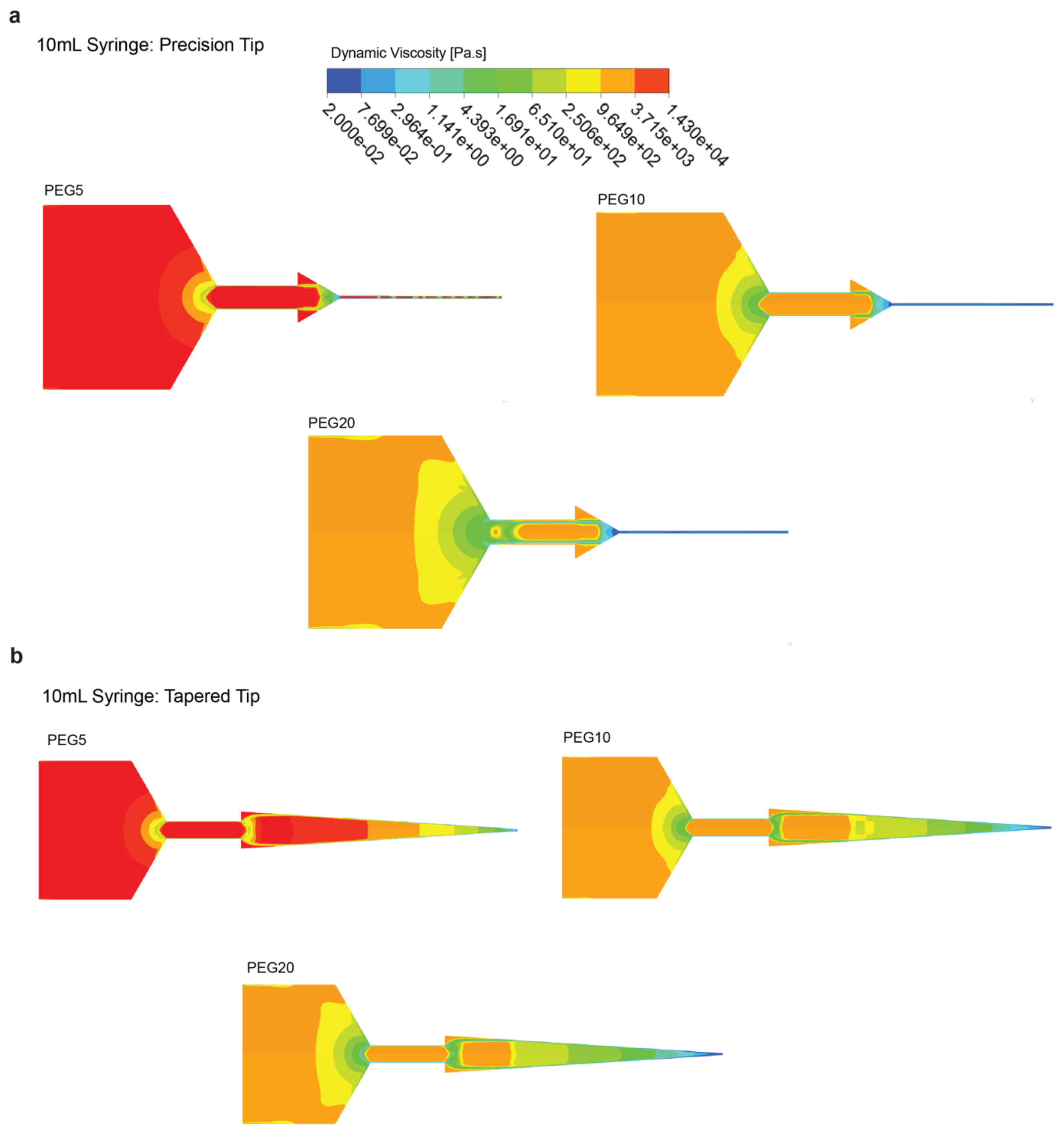


**Fig. S8.**
**Dynamic viscosity simulations of PEG5, PEG10, and PEG20 HMP printing using 10 mL syringes.**

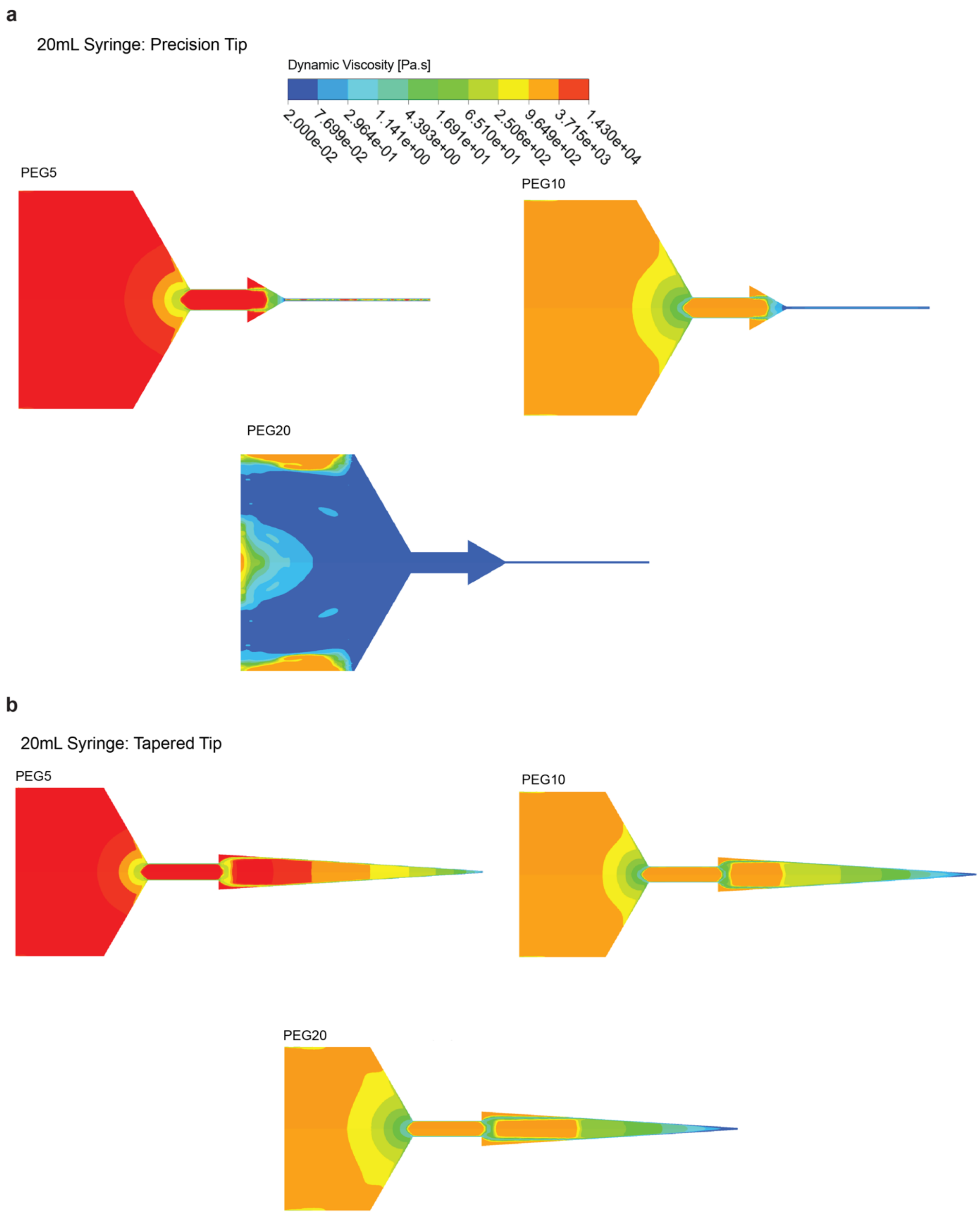


**Fig. S9.**

**Dynamic viscosity simulations of PEG5, PEG10, and PEG20 HMP printing using 20 mL syringes.**

| **Model** | **Equation** | **$R^2$** |
|---|---|---|
| Power-Law | $\eta = K\dot{\gamma}^{n-1}$ | 0.834 |
| Herschel-Bulkley | $\eta = \eta_0 + K\dot{\gamma}^{n}$ | 0.832 |
| Carreau | $\eta = (\mu - \mu_2)/(1+\lvert K\dot{\gamma}\rvert^{1-n})$ | 0.994 |

**Table S1.**

**Model choice for Fluent simulations based on $R^2$ value of fitting shear-rate sweep data from rheology.**

**Movie S1.**
Video showing smooth and consistent extrusion of an HMP filament in a printable condition.

**Movie S2.**
Video showing inconsistent and burst extrusion of an HMP filament in a non-printable condition.
